# Longitudinal Serum and Cerebrospinal Fluid S100B Concentrations After Aneurysmal Subarachnoid Hemorrhage: An Exploratory Comparison of Historical Cohorts With and Without Cerebrolysin Exposure

**DOI:** 10.3390/jcm15218734

**Published:** 2026-11-09

**Authors:** Małgorzata Burzyńska, Adrianna Lebiedzińska, Jarosław Kędziora, Barbara Dragan, Bogusława Lechowicz-Głogowska, Barbara Adamik, Marek Wełna, Waldemar Goździk

**Affiliations:** 1 Clinical Department of Anesthesiology and Intensive Therapy, Faculty of Medicine, Wroclaw Medical University, 50-367 Wroclaw, Poland; malgorzata.burzynska@umw.edu.pl (M.B.); jaroslaw.kedziora@umw.edu.pl (J.K.); barbara.dragan@umw.edu.pl (B.D.); boguslawa.lechowicz-glogowska@umw.edu.pl (B.L.-G.); barbara.adamik@umw.edu.pl (B.A.); marek.welna@umw.edu.pl (M.W.); 2 Clinical Department of Anesthesiology and Intensive Therapy, University Clinical Hospital in Wroclaw, 50-556 Wroclaw, Poland; adrianna.lebiedzinska@usk.wroc.pl

**Keywords:** aneurysmal subarachnoid hemorrhage, cerebrolysin, protein S100B

## Abstract

**Background/Objectives:** Cerebrolysin (CBL) has been proposed to exert neuroprotective and neurotrophic effects, but its biological effects after aneurysmal subarachnoid hemorrhage (aSAH) remain uncertain. We investigated longitudinal serum and cerebrospinal fluid (CSF) S100 calcium-binding protein B (S100B) profiles according to CBL exposure. **Methods:** This retrospective, single-center exploratory cohort study included 67 patients with aSAH (CBL, *n* = 26; non-CBL, *n* = 41). S100B was measured in serum and, when CSF access was clinically available, in CSF on days 1, 3, 5, 7, and 10. In the CBL cohort, treatment was initiated before the first study sample, which was obtained approximately 12 h after the first dose on average. Accordingly, day 1 was considered an early post-exposure measurement rather than a pre-treatment baseline. Longitudinal serum and CSF S100B concentrations were analyzed using linear mixed-effects models with patient-specific random intercepts, including CBL exposure, study day, and their interaction. A sensitivity analysis was performed after exclusion of day 1. **Results:** A significant overall between-cohort difference in serum S100B was observed across the study period (geometric mean ratio [GMR] 0.028, 95% CI 0.013–0.057; *p* < 0.001), with no significant CBL-by-day interaction (*p* = 0.218). The association persisted after exclusion of day 1 (GMR 0.023, 95% CI 0.011–0.049; *p* < 0.001). No significant overall between-cohort difference was observed for CSF S100B (GMR 0.542, 95% CI 0.185–1.584; *p* = 0.263). The CSF-to-serum ratio was higher in the CBL cohort on days 1, 3, 5, and 7 but not on day 10. No significant between-group differences were observed in delayed cerebral ischemia (DCI), TCD-detected cerebral vasospasm, mortality, or functional outcomes. **Conclusions:** CBL exposure was associated with persistently lower serum S100B concentrations, whereas no corresponding overall between-cohort difference was observed in CSF. These findings indicate a differential association of CBL exposure with serum and CSF S100B profiles; however, because CBL exposure was completely confounded by treatment era, the observed associations cannot establish a causal treatment effect. Prospective, contemporaneous controlled studies are required to determine whether CBL directly influences S100B dynamics after aSAH.

## 1. Introduction

Aneurysmal subarachnoid hemorrhage (aSAH) is a severe cerebrovascular disease associated with substantial neurological and systemic morbidity [1,2,3]. Despite advances in early aneurysm securing and neurocritical care, secondary brain injury evolving during the days following hemorrhage remains a major determinant of clinical outcomes [4,5,6]. This process involves multiple interacting mechanisms, including impaired cerebral autoregulation, endothelial and microvascular dysfunction, microvascular thrombosis, neuroinflammation, oxidative stress, cortical spreading depolarizations, and cerebral vasospasm (CV) [7,8,9,10]. These pathophysiological processes are initiated early after aneurysm rupture, when extravasated blood within the subarachnoid space triggers a complex cascade of cellular and vascular responses. Delayed cerebral ischemia (DCI), which reflects the clinical consequences of these interacting mechanisms, remains one of the most important potentially modifiable complications after aSAH [11]. This evolving understanding has shifted increasing research attention toward the mechanisms initiated during the early brain injury phase and their contribution to subsequent DCI and secondary brain injury.

S100 calcium-binding protein B (S100B) is an astroglial protein that can be detected in cerebrospinal fluid (CSF) and peripheral blood following acute brain injury [12,13,14,15]. Elevated and persistently increased serum S100B concentrations after aSAH have been associated with greater neurological injury and unfavorable clinical outcomes [16,17,18,19,20].

However, serum S100B concentrations may reflect not only the extent of cerebral injury but also the transport and clearance of the protein across the blood–brain and CSF–blood barriers. Consequently, discordant changes in CSF and serum S100B concentrations may provide complementary information about the biological processes underlying secondary brain injury that cannot be captured by either compartment alone.

Cerebrolysin^®^ (CBL; EVER Neuro Pharma GmbH, Unterach, Austria) is a peptide-based preparation derived from enzymatically hydrolyzed porcine brain proteins. It has been proposed to exert neurotrophic, anti-inflammatory, antioxidant, and neuroprotective effects [21,22,23]. Experimental and clinical studies have suggested potential effects on mechanisms relevant to secondary brain injury; however, evidence regarding its biological effects after aSAH remains limited [24,25,26]. In particular, it remains unclear whether CBL exposure is associated with differences in the longitudinal profile or compartmental distribution of S100B after aSAH.

Given the involvement of neuroinflammation, endothelial dysfunction, and blood–brain barrier (BBB) disruption in secondary brain injury after aSAH, differences in S100B dynamics according to CBL exposure could provide exploratory insight into potential biological associations of CBL with post-aSAH brain injury. Nevertheless, the relationship between CBL exposure and serial S100B concentrations in serum and CSF after aSAH has not been adequately investigated.

Therefore, the present study investigated longitudinal S100B profiles in serum and, when CSF was clinically accessible, CSF S100B profiles in patients with aSAH who received CBL and in patients treated without CBL. The primary aim of this single-center exploratory cohort study was to characterize longitudinal differences in serum and CSF S100B concentrations, and in the CSF-to-serum S100B ratio, according to CBL exposure.

Secondary aims were to explore associations between longitudinal S100B concentrations, DCI and clinical outcomes. Sensitivity analyses stratified by aneurysm-securing modality and CSF drainage route were performed to assess the robustness of the principal biomarker findings. Given the observational design of the study and the potential for confounding, particularly by treatment era, the study was intended primarily to characterize associations between CBL exposure and S100B dynamics rather than to establish the clinical efficacy or causal biological effects of CBL.

## 2. Materials and Methods

### 2.1. Study Design and Patient Selection

This retrospective, single-center exploratory cohort study included adult patients with aSAH who were treated in the Neurointensive Care Unit of the University Clinical Hospital in Wroclaw, Poland. The study involved a retrospective analysis of prospectively collected clinical and biological data from two predefined treatment periods: from 6 March 2014 to December 2017 and March 2023 to December 2024.

S100B measurements were obtained within two separate prospective research projects and were not routinely available during the intervening period 2018–2022. Accordingly, the study periods were determined by the availability of comparable clinical and biomarker data rather than continuous recruitment.

Adult patients with aSAH admitted within 24 h of symptom onset were eligible for inclusion if the ruptured aneurysm was secured by microsurgical clipping or endovascular coiling within 24 h of hospital admission, paired CSF and serum samples were available for S100B measurement at the predefined study time points, and the clinical data required for the planned analyses were available. The availability of measurements varied across study days; missing observations were not imputed.

**Figure 1:**
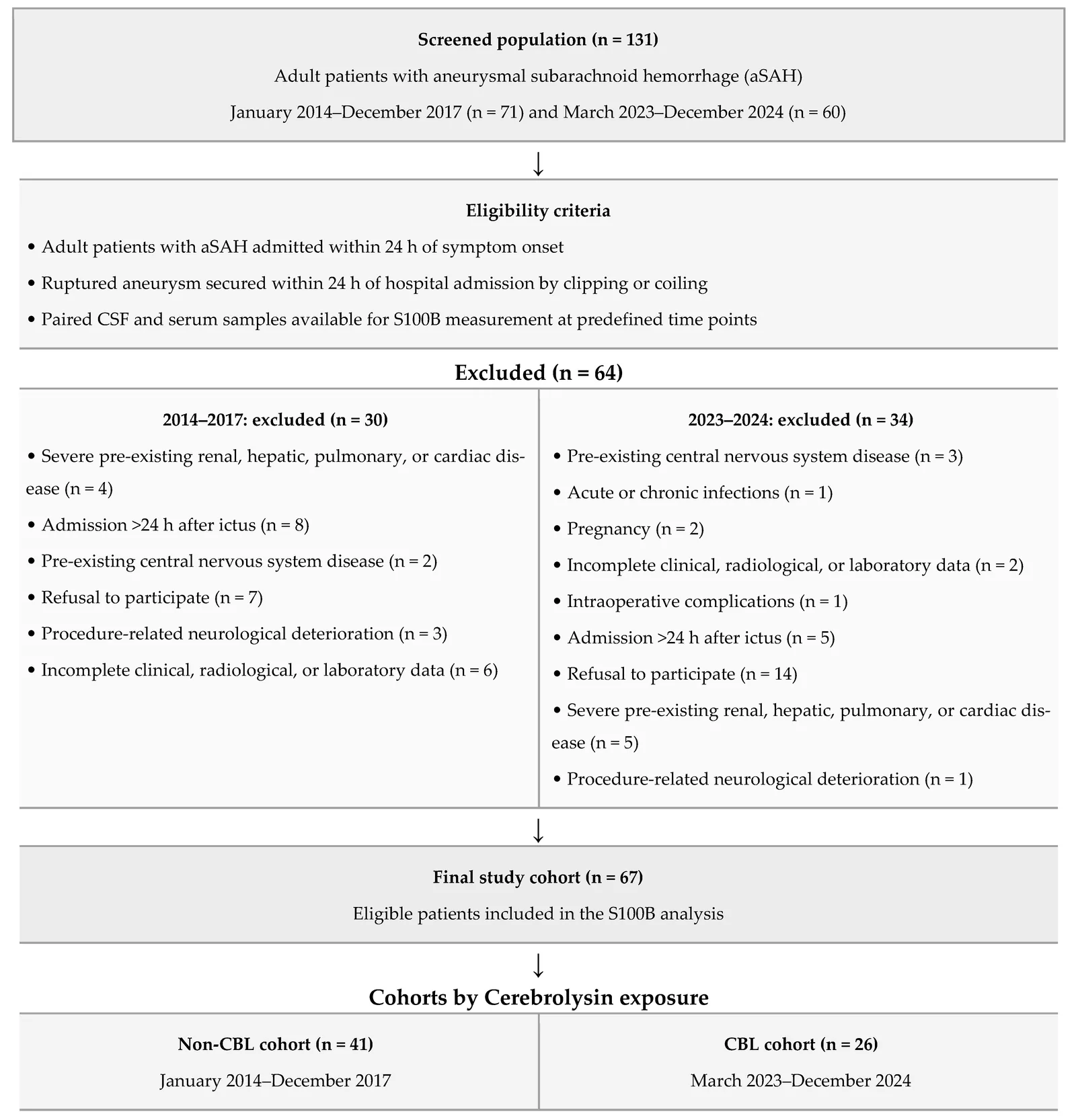
Flowchart of patient selection for the comparison of CSF and serum S100B concentrations between patients with and without Cerebrolysin exposure. aSAH, aneurysmal subarachnoid hemorrhage; CBL, Cerebrolysin; CSF, cerebrospinal fluid.

Exclusion criteria included pre-existing central nervous system disease, including previous stroke, traumatic brain injury, or aSAH; severe pre-existing renal, hepatic, pulmonary, or cardiac disease; pregnancy; admission more than 24 h after ictus; refusal to participate; procedure-related neurological deterioration or intraoperative complications; and incomplete clinical, radiological, or laboratory data required for analysis. Acute or chronic infection was an additional exclusion criterion in the 2023–2024 screening cohort. Period-specific exclusions are shown in Figure 1.

Patients were assigned into two cohorts according to treatment period and corresponding CBL exposure. The non-CBL cohort comprised patients treated between January 2014 and December 2017, before the introduction of CBL into routine institutional practice. The CBL cohort comprised patients treated between March 2023 and December 2024.

Because CBL exposure was linked to treatment period, the effects of CBL exposure and calendar period could not be evaluated independently. All between cohort comparisons were therefore interpreted as associative rather than causal.

### 2.2. Cerebrolysin (CBL) Treatment

Patients in the CBL cohort received CBL at a dose of 30 mL intravenously once daily, initiated on day 1 after aSAH, according to the institutional treatment protocol. The inclusion of CBL in this protocol was informed by European Neurorehabilitation Recommendations supporting its use as an adjunctive treatment during early recovery after moderate-to-severe acute ischemic stroke [27], although CBL is not specifically recommended in current aSAH guidelines. CBL was administered in addition to standard neurocritical care and aneurysm-directed treatment. In the CBL cohort, treatment was initiated before the first study sample was collected. The interval between the first CBL administration and day-1 sampling was approximately 12 h on average. Accordingly, day-1 S100B concentrations were considered early post-exposure measurements rather than pre-treatment baseline values.

Patients in the non-CBL cohort received standard care according to the institutional protocol applicable during the corresponding treatment period but did not receive CBL.

### 2.3. Clinical Management

The diagnosis of aSAH was established by non-contrast cranial computed tomography (CT) and confirmed by vascular imaging using CT angiography (CTA) and/or digital subtraction angiography (DSA). Patients received neurocritical care according to institutional protocols and the European Stroke Organization, American Heart Association/American Stroke Association, and Neurocritical Care Society recommendations applicable during the respective study periods [28,29,30,31]. The guideline framework and institutional management in the two treatment periods are summarized in Table 1. Although the main components of care were similar, differences in aneurysm-securing modality and CSF drainage practices remained potential treatment-era confounding.

Patients presenting with acute severe hydrocephalus were managed primarily with external ventricular drainage (EVD). In selected patients, lumbar drainage (LD) was used to facilitate clearance of subarachnoid blood degradation products and as part of the institutional strategy aimed at reducing the risk of DCI. Lumbar drains were typically placed immediately after endovascular aneurysm treatment or within 24 h thereafter. In patients with communicating hydrocephalus, intermittent lumbar CSF drainage was performed when clinically indicated, with approximately 10–20 mL of CSF drained per session to reduce elevated CSF pressure. In patients without hydrocephalus, controlled continuous lumbar drainage was performed using a controlled drainage strategy, generally at a rate of 5–8 mL/h, with adjustment up to 10 mL/h depending on the subarachnoid blood burden and the clinical condition. Drainage usually continued for approximately 5–8 days. Patients with acute severe hydrocephalus were generally managed with EVD alone and did not routinely undergo concomitant lumbar drainage. The choice of CSF diversion strategy was based on the presence and severity of hydrocephalus, the distribution of subarachnoid blood, and the treating team’s clinical assessment.

Routine management included oral or enteral nimodipine, maintenance of euvolemia and adequate arterial blood pressure, and careful fluid and electrolyte management. Hemodynamic monitoring included continuous invasive arterial blood pressure measurement, central venous pressure monitoring, hourly urine output and fluid balance monitoring. Vasopressor and, when clinically indicated, inotropic support were individualized according to clinical and hemodynamic status. Prophylactic hypervolemia was not routinely used. Follow-up CT was performed within 24 h after aneurysm securing. Additional CT, CTA, and/or MRI was obtained as clinically indicated.

Multimodal neuromonitoring was used in both treatment periods and included serial neurological assessment, daily TCD (Doppler-BoxX, Compumedics Germany GmbH, Singen, Germany), intracranial pressure (ICP) using Codman (IntegraLifeSciences, Princeton, NJ, USA) or Camino sensors (Integra LifeSciences, Plainsboro, NJ, USA) and cerebral perfusion pressure (CPP) monitoring when clinically indicated, and near-infrared spectroscopy (NIRS)-based regional cerebral oxygenation monitoring using NIRS (FORE-SIGHT Elite, CAS Medical Systems, Branford, CT, USA). Continuous invasive arterial pressure monitoring supported CPP assessment in patients undergoing ICP monitoring.

Cerebral vasospasm (CV) was assessed using daily TCD ultrasonography and diagnosed according to established TCD criteria and recommendations applicable during the respective study periods [32].

DCI was defined according to established consensus criteria as the occurrence of a new focal neurological impairment and/or a decrease in the level of consciousness that could not be explained by other causes, including hydrocephalus, seizures, metabolic disturbances, infection, hypoxemia, hemodynamic instability, or medication effects. New cerebral ischemic lesions were assessed using CT and/or MRI when clinically indicated [33].

In patients with clinically suspected or confirmed DCI, induced hypertension with maintenance of euvolemia was used as first-line hemodynamic therapy when not contraindicated. Endovascular rescue treatment, including balloon angioplasty and/or intra-arterial administration of nimodipine, was considered in patients with persistent neurological deterioration or an insufficient response to hemodynamic augmentation.

All patients received continuous cardiorespiratory monitoring and supportive treatment tailored to their clinical condition. Respiratory function was routinely monitored. Patients requiring mechanical ventilation were generally ventilated using a BiLevel mode, with the lowest feasible FiO_2_ required to maintain peripheral oxygen saturation (SpO_2_) at least 94% and a target arterial carbon dioxide tension (PaCO_2_) of 35–45 mmHg. Sedation, predominantly with propofol, was titrated according to the Richmond Agitation–Sedation Scale (RASS). Multimodal analgesia was provided and adjusted using the Visual Analogue Scale (VAS) in responsive patients or a behavioral pain assessment scale in patients unable to self-report pain. Pharmacological thromboprophylaxis was initiated after aneurysm securing when not contraindicated.

Infection prevention measures included hand hygiene, appropriate catheter and airway care, and minimization of unnecessary invasive devices. Early enteral nutrition was initiated whenever feasible. Electrolyte, metabolic, acid–base, glycemic, and other systemic disturbances were monitored and corrected as indicated. Temperature management and organ-specific supportive treatment were individualized.

### 2.4. Clinical and Radiological Variables

Recorded variables included demographic characteristics, premorbid medical history, neurological status at admission, aneurysm location, intracerebral hemorrhage (ICH), aneurysm-securing modality, CSF diversion strategy, decompressive craniectomy, TCD-detected CV, and DCI during the ICU stay.

Disease severity at admission was characterized using APACHE II, the Glasgow Coma Scale (GCS), the motor GCS component (m-GCS), the Full Outline of UnResponsiveness (FOUR) score, Hunt–Hess grade, and World Federation of Neurosurgical Societies (WFNS) grade [34,35].

The radiological severity of the initial hemorrhage and early cerebral edema was assessed on admission non-contrast CT. Early cerebral edema was quantified using the Subarachnoid Hemorrhage Early Brain Edema Score (SEBES), a 0–4-point radiographic scale based on sulcal effacement and loss of gray–white matter differentiation at two predefined axial CT levels in each cerebral hemisphere [36].

The burden of subarachnoid hemorrhage was assessed using the modified Fisher scale (mFS), which incorporates the thickness of subarachnoid blood and the presence of intraventricular hemorrhage and ranges from 0 to 4 [37]. DCI risk was additionally characterized using the VASOGRADE classification: Green (WFNS I–II and mFS 1–2), Yellow (WFNS I–III and mFS 3–4), or Red (WFNS IV–V irrespective of mFS) [38].

Functional outcome was assessed at hospital discharge and at 3 months using the modified Rankin Scale (mRS), ranging from 0 (no symptoms) to 6 (death). A favorable functional outcome was prespecified as mRS 0–2, whereas mRS 3–6 was classified as an unfavorable functional outcome for dichotomous analyses [39,40].

### 2.5. Cerebrospinal Fluid and Serum Sampling

Availability of paired serum and CSF samples was not required for study inclusion. Serum samples were collected according to the predefined study schedule, whereas CSF samples were obtained only when CSF access was clinically indicated and available. The first samples were obtained within 24 h of symptom onset, followed by sampling on study days 3, 5, 7, and 10. CSF samples were obtained through an external ventricular drain (EVD) or lumbar drain (LD) when clinically indicated. Venous blood samples were collected through a central venous catheter. Whenever both compartments were sampled, CSF and blood were collected simultaneously to enable paired assessment of S100B concentrations. At each sampling time point, 4.5 mL of available CSF and 4.5 mL of blood were collected.

In the CBL cohort, treatment was initiated before collection of the first study sample, and the interval between the first CBL administration and day-1 sampling was approximately 12 h on average. Consequently, day-1 S100B measurements were considered early post-exposure measurements rather than pre-treatment baseline values.

Immediately after collection, samples were centrifuged at 4000 rpm for 10 min at 4 °C according to a standardized laboratory protocol. Serum and CSF supernatant were subsequently aliquoted and stored at −80 °C until analysis. To minimize pre-analytical variability, aliquoting was performed before freezing, and all samples underwent only a single freeze–thaw cycle before S100B measurement.

Before analysis, all samples were assessed for pre-analytical quality and compliance with the predefined processing and storage requirements. Samples were excluded if they were incorrectly or ambiguously labeled, insufficient in volume for the planned assay, showed significant hemolysis, had been stored or transported under conditions inconsistent with the predefined protocol, had remained in prolonged contact with the blood clot, or showed evidence of compromised specimen integrity or other features potentially affecting analytical reliability. Only samples meeting all predefined quality criteria were included in the final S100B analysis.

During the 2014–2017 study period, a total of 230 biological samples were collected. Of these, 31 samples were excluded because they did not meet the predefined quality criteria, leaving 199 samples for S100B analysis, including 154 serum samples and 45 CSF samples. During the 2023–2024 study period, 166 biological samples were collected. Of these, 13 were excluded based on the same predefined quality criteria, leaving 153 samples for analysis, including 100 serum samples and 53 CSF samples.

Samples from both cohorts were analyzed in the same laboratory, using the same analytical method, and by the same experienced laboratory operator.

### 2.6. S100B Measurement

S100B concentrations in CSF and serum were quantified using a commercially available Human S100B DuoSet enzyme-linked immunosorbent assay (ELISA) kit (catalog no. DY1820-05; R&D Systems, Minneapolis, MN, USA), according to the manufacturer’s instructions.

The stated calibration range of the assay was 0.0469–3.0 ng/mL The lower calibration limit of 0.0469 ng/mL referred to the concentration in the original sample before dilution. S100B concentrations were derived from the assay calibration curve. All samples were analyzed in duplicate with appropriate assay controls.

Samples with S100B concentrations exceeding the upper calibration limit were additionally diluted 1:10 and reanalyzed. Final concentrations were corrected for the dilution factor and are reported as concentrations in the original undiluted sample.

Values below the lower calibration limit were retained in the primary exploratory analysis. However, because quantitative precision below the calibrated range may be limited, the robustness of the serum S100B findings was additionally assessed in sensitivity analyses using alternative approaches to handling values below 0.0469 ng/mL.

Across all sampling days, 56 of 100 available serum measurements in the CBL cohort (56.0%) were below the lowest calibration standard of 0.0469 ng/mL, compared with none of 154 available serum measurements in the non-CBL cohort. These proportions were calculated using available measurements, with missing observations excluded from the denominator. Below-range values were retained in the primary exploratory analysis; however, their quantitative accuracy remains uncertain.

At collection, specimens were labeled using coded patient identifiers and sampling time points. Personnel performing the S100B assays were blinded to patient identity and to the correspondence between serum and CSF specimens. After completion of the laboratory measurements, coded samples were linked to the clinical database, and serum and CSF specimens obtained from the same patient at the same predefined time point were identified as paired samples for the relevant analyses.

### 2.7. Definition of the CSF-to-Serum S100B Ratio

For each paired measurement, the CSF-to-serum S100B ratio was calculated by dividing the CSF S100B concentration by the corresponding serum S100B concentration (CSF S100B concentration/serum S100B concentration). The calculation was adapted from the CSF-to-plasma biomarker quotient approach described by Kapapa et al. [41]. Ratios were calculated only when paired CSF and serum measurements obtained at the same time point were available.

Because the distribution of CSF-to-serum S100B ratios was markedly right-skewed, individual ratios are displayed on a logarithmic vertical axis in the plot. The plotted values are raw ratios rather than a separate panel of natural-log-transformed ratios. Time-specific between-cohort comparisons were performed on raw ratios using the Mann–Whitney U test, as described in Section 2.9. Only same-day pairs with strictly positive concentrations in both compartments were included [42,43].

Time-specific between-group inferential comparisons were performed on the raw ratios as described in Section 2.9. The natural-log transformation was introduced as part of the statistical methodology of the present study and was not derived from the approach described by Kapapa et al. [41]. Only paired measurements with S100B concentrations greater than zero in both compartments were included in the log-ratio analyses because the logarithmic ratio was undefined when either concentration was zero.

### 2.8. Ethics and Reporting

The original prospective data-collection protocols were conducted in accordance with the Declaration of Helsinki and were reviewed and approved by the Ethics Committee of Wroclaw Medical University (approval Nos. KB-134/2014 and KB-191/2023, dated 6 March 2014 and 9 March 2023, respectively). Written informed consent was obtained from all participants or their legally authorized representatives before enrollment in the respective prospective protocols. Based on the study protocol and the previously issued ethical approvals, the Bioethics Committee subsequently issued Certificate No. 332/2026 in July 2026.

The present study involved a retrospective analysis of prospectively collected observational data and was reported in accordance with the Strengthening the Reporting of Observational Studies in Epidemiology (STROBE) guidelines.

### 2.9. Statistical Analysis

Continuous and ordinal variables are presented as medians with interquartile ranges (IQRs), and categorical variables as *n* (%). Between-group comparisons were performed using the two-sided Mann–Whitney U test for continuous or ordinal variables and Fisher’s exact test for categorical variables; the VASOGRADE distribution was compared using the Fisher–Freeman–Halton exact test. For very small subgroup comparisons without ties, exact Wilcoxon rank-sum *p* values were used; otherwise, asymptotic *p* values with continuity correction were applied.

Because S100B concentrations were right-skewed, serum and CSF values were transformed as ln(S100B + c) before linear mixed-effects modeling, where c corresponded to half of the smallest positive value in the respective dataset (0.0008 ng/mL for serum and 0.03 ng/mL for CSF). Models were fitted by restricted maximum likelihood with patient-specific random intercepts and fixed effects for CBL exposure, categorical study day, and the CBL-by-day interaction. Omnibus CBL-by-day and time effects were evaluated using joint Wald chi-square tests. When the CBL-by-day interaction was non-significant, an additive model was used to estimate the overall between-cohort difference, expressed as a geometric mean ratio (GMR) with Wald 95% confidence intervals. Because day 1 represented an early post-exposure measurement in the CBL cohort, sensitivity analyses were additionally performed after exclusion of day 1.

Time-specific comparisons of serum S100B, CSF S100B, and the CSF-to-serum ratio were performed using the Mann–Whitney U test, with Benjamini–Hochberg (BH) adjustment for multiple comparisons across the five study time points. Ratio analyses included only same-day paired measurements with CSF S100B > 0 and serum S100B > 0.

Additional sensitivity analyses were performed according to aneurysm-securing modality, CSF drainage route, and alternative substitution of serum S100B values below the lower calibration boundary. Associations of longitudinal serum and CSF S100B concentrations with DCI, functional outcome, and in-hospital mortality were evaluated using mixed-effects models. Within each CBL stratum, models included outcome status and categorical study day; pooled models additionally included CBL exposure and a CBL-by-outcome interaction. Missing observations were not imputed.

Additional severity-adjusted linear mixed-effects models included APACHE II and WFNS grade as covariates, with patient-specific random intercepts and fixed effects for CBL exposure and categorical study day. Extended sensitivity models additionally adjusted for CSF drainage route and aneurysm-securing modality. The CBL effect was expressed as a geometric mean ratio (GMR). Confidence intervals and *p* values were obtained using the procedures specified for each model in the Supplementary Materials. Sensitivity analyses also examined alternative substitutions for serum S100B values below the lower calibration boundary.

Exploratory Spearman correlations among patient-level S100B-derived indices were performed using pairwise complete observations, with BH adjustment of unique pairwise correlation *p* values within each matrix and are reported in the Supplementary Materials. All tests were two-sided. A *p* value < 0.05 was considered statistically significant, and BH-adjusted *p* values < 0.05 were considered significant for multiple comparisons. Secondary, subgroup, and sensitivity analyses were considered exploratory. Statistical analyses were performed using R version 4.5.2 (R Foundation for Statistical Computing, Vienna, Austria); linear mixed-effects models were fitted using the lme4 package.

## 3. Results

### 3.1. Patient Characteristics

A total of 67 patients were included, including 26 in the CBL cohort and 41 in the non-CBL cohort. Patient selection and cohort formation are presented in Figure 1, and baseline demographic, clinical, and treatment-related characteristics are summarized in Table 2. Non-significant between-cohort comparisons should not be interpreted as evidence of baseline balance. Differences in patient characteristics and management remain potential sources of confounding in this historical-cohort comparison. Endovascular coiling was more frequent in the CBL cohort (84.6% vs. 63.4%), although the difference was not statistically significant (*p* = 0.094). Lumbar drainage was significantly more common in the CBL cohort (42.3% vs. 12.2%; *p* = 0.008). No significant between-group differences were observed in DCI, TCD-detected vasospasm, mortality, or functional outcomes.

**Table 2:** Patient Characteristics According to CBL Exposure.

Characteristic	Overall (*N* = 67)	CBL (*N* = 26)	Non-CBL (*N* = 41)	*p* Value
Demographics				
Age, years	56.0 (48.0–67.0)	54.0 (41.5–60.8)	60.0 (50.0–70.0)	0.124
Female sex, *n* (%)	40 (59.7%)	15 (57.7%)	25 (61.0%)	0.804
BMI, kg/m^2^	26.0 (23.1–29.7)	24.7 (23.0–27.3)	27.6 (23.4–30.4)	0.200
Comorbidities				
Hypertension, *n* (%)	40 (59.7%)	12 (46.2%)	28 (68.3%)	0.081
Current smoking, *n* (%)	41 (61.2%)	17 (65.4%)	24 (58.5%)	0.616
Diabetes mellitus, *n* (%)	3 (4.5%)	3 (11.5%)	0 (0.0%)	0.054
History of heart disease *n* (%)	9 (13.4%)	3 (11.5%)	6 (14.6%)	1.000
Admission severity				
APACHE II	16.0 (11.0–24.0)	18.5 (12.2–29.8)	15.0 (10.0–20.0)	0.069
m-GCS	5.0 (4.0–6.0)	5.0 (4.0–6.0)	6.0 (4.0–6.0)	0.752
GCS	13.0 (7.0–14.5)	13.0 (6.0–15.0)	13.0 (7.0–14.0)	0.906
FOUR score	14.0 (8.0–16.0)	14.0 (8.0–16.0)	15.0 (11.0–16.0)	0.455
SEBES	2.0 (0.0–3.0)	2.0 (2.0–3.0)	2.0 (0.0–3.0)	0.326
Hunt–Hess grade	3.0 (2.0–4.0)	3.0 (2.0–5.0)	3.0 (2.0–4.0)	0.926
modified Fisher grade	4.0 (3.0–4.0)	4.0 (3.0–4.0)	3.0 (3.0–4.0)	0.488
WFNS grade	3.0 (1.0–5.0)	2.5 (1.0–5.0)	3.0 (1.0–4.0)	0.765
VASOGRADE				0.829
Red	27 (40.3%)	11 (42.3%)	16 (39.0%)	
Yellow	32 (47.8%)	13 (50.0%)	19 (46.3%)	
Green	8 (11.9%)	2 (7.7%)	6 (14.6%)	
Intracerebral hematoma (ICH)	19 (28.4%)	7 (26.9%)	12 (29.3%)	1.000
Aneurysm location				
Anterior circulation	56 (83.6%)	23 (88.5%)	33 (80.5%)	0.508
Posterior circulation	11 (16.4%)	3 (11.5%)	8 (19.5%)	0.508
Treatment				
Endovascular coiling	48 (71.6%)	22 (84.6%)	26 (63.4%)	0.094
Surgical clipping	19 (28.4%)	4 (15.4%)	15 (36.6%)	0.094
Surgical treatment for ICH	12 (17.9%)	6 (23.1%)	6 (14.6%)	0.515
Decompressive craniectomy	10 (14.9%)	6 (23.1%)	4 (9.8%)	0.169
Lumbar drainage	16 (23.9%)	11 (42.3%)	5 (12.2%)	0.008
External ventricular drainage	18 (26.9%)	6 (23.1%)	12 (29.3%)	0.778
Clinical course and outcomes				
TCD-detected vasospasm	41 (61.2%)	14 (53.8%)	27 (65.9%)	0.441
Delayed cerebral ischemia	35 (52.2%)	15 (57.7%)	20 (48.8%)	0.617
ICU length of stay, days	13.0 (8.5–19.0)	13.0 (9.2–18.8)	12.0 (8.0–19.0)	0.801
Hospital length of stay, days	19.0 (14.0–28.5)	20.0 (14.2–34.5)	19.0 (13.0–28.0)	0.467
In-hospital mortality	17 (25.4%)	6 (23.1%)	11 (26.8%)	0.781
mRS at discharge	4.0 (1.5–5.5)	4.0 (2.2–5.0)	3.0 (1.0–6.0)	0.436
mRS at 3 months	3.0 (1.0–5.5)	3.0 (1.2–5.0)	2.0 (1.0–6.0)	0.543

Data are presented as median (IQR) for continuous/ordinal variables and *n* (%) for categorical variables. Between-group comparisons were performed using the Mann–Whitney U test for continuous/ordinal variables and Fisher’s exact test for categorical variables. The VASOGRADE distribution was compared using the Fisher–Freeman–Halton exact test. All variables shown had complete data for all 67 patients. Abbreviations: APACHE II, Acute Physiology and Chronic Health Evaluation II; BMI, body mass index; CBL, Cerebrolysin; FOUR, Full Outline of UnResponsiveness; GCS, Glasgow Coma Scale; ICH, intracerebral hematoma; ICU, intensive care unit; LD, lumbar drainage; EVD, external ventricular drainage; m-GCS, motor Glasgow Coma Scale; mRS, modified Rankin Scale; SEBES, Subarachnoid Hemorrhage Early Brain Edema Score; TCD, transcranial Doppler; WFNS, World Federation of Neurosurgical Societies.

### 3.2. Serum and CSF S100B Concentrations According to CBL Exposure

**Figure 2:**
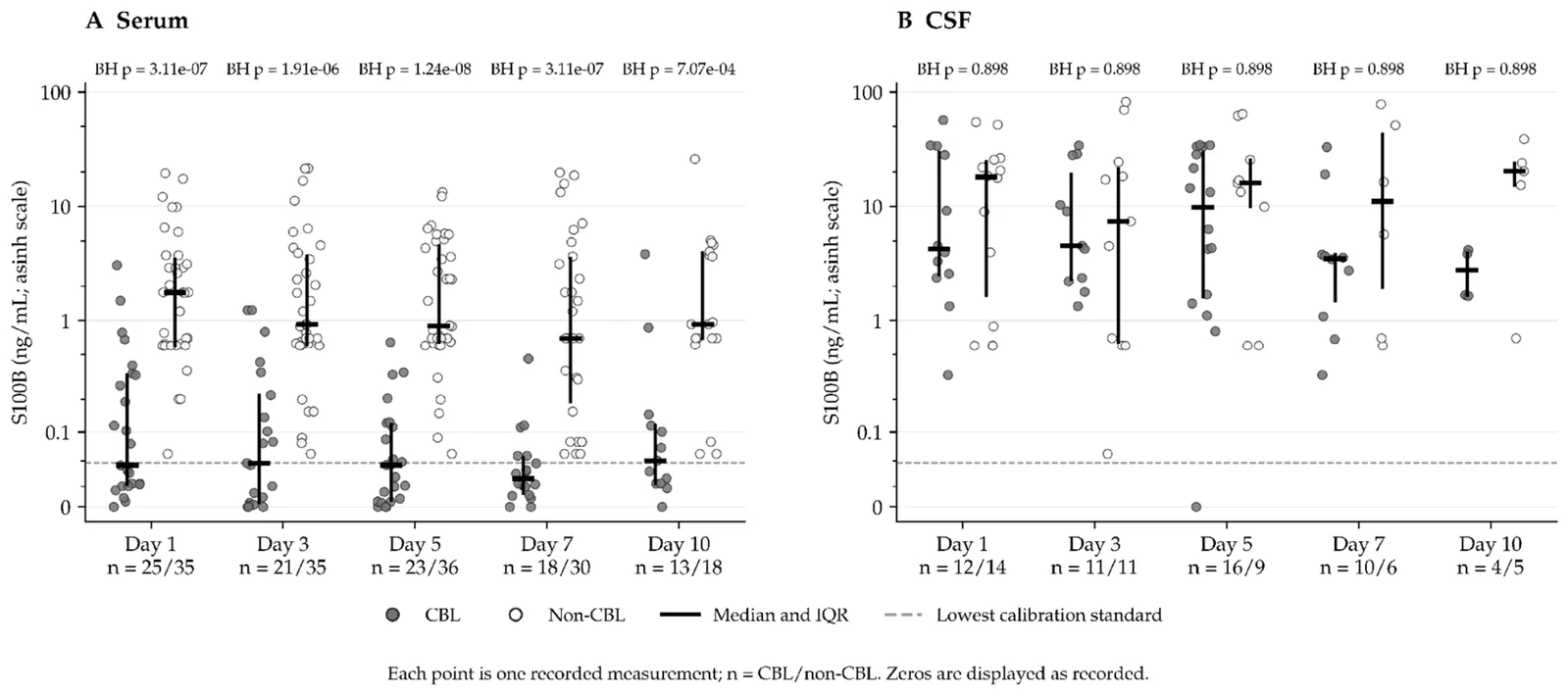
Individual serum (**A**) and CSF (**B**) S100B concentrations on days 1, 3, 5, 7 and 10 in historical cohorts with and without Cerebrolysin exposure. Filled circles represent CBL and open circles non-CBL measurements. Horizontal displacement improves visibility. Black horizontal marks indicate medians; vertical lines indicate IQRs. The asinh scale is approximately linear near zero and logarithmic at higher concentrations; tick labels show ng/mL Its transition parameter is 0.0469 ng/mL, also indicated by the dashed line marking the lowest calibration standard. Recorded zeros and positive below-range values are displayed without substitution; their display does not validate quantitative accuracy. Missing observations are omitted. Sample sizes are shown as CBL/non-CBL. Between-cohort two-sided Mann–Whitney U tests use automatic exact/asymptotic selection, with BH correction separately across five days per compartment. CBL, Cerebrolysin; CSF, cerebrospinal fluid; IQR, interquartile range; BH, Benjamini–Hochberg.

Significant between-group differences in serum S100B concentrations were observed at all study time points, with all comparisons remaining significant after Benjamini–Hochberg correction (Table 3; Figure 2A).

In contrast, CSF S100B concentrations did not differ significantly between cohorts at any individual time point (Table 4; Figure 2B). Because CBL treatment was initiated before collection of the first study sample, day-1 measurements in the CBL cohort represented early post-exposure values rather than pre-treatment baseline concentrations.

### 3.3. CSF-to-Serum S100B Ratio

**Figure 3:**
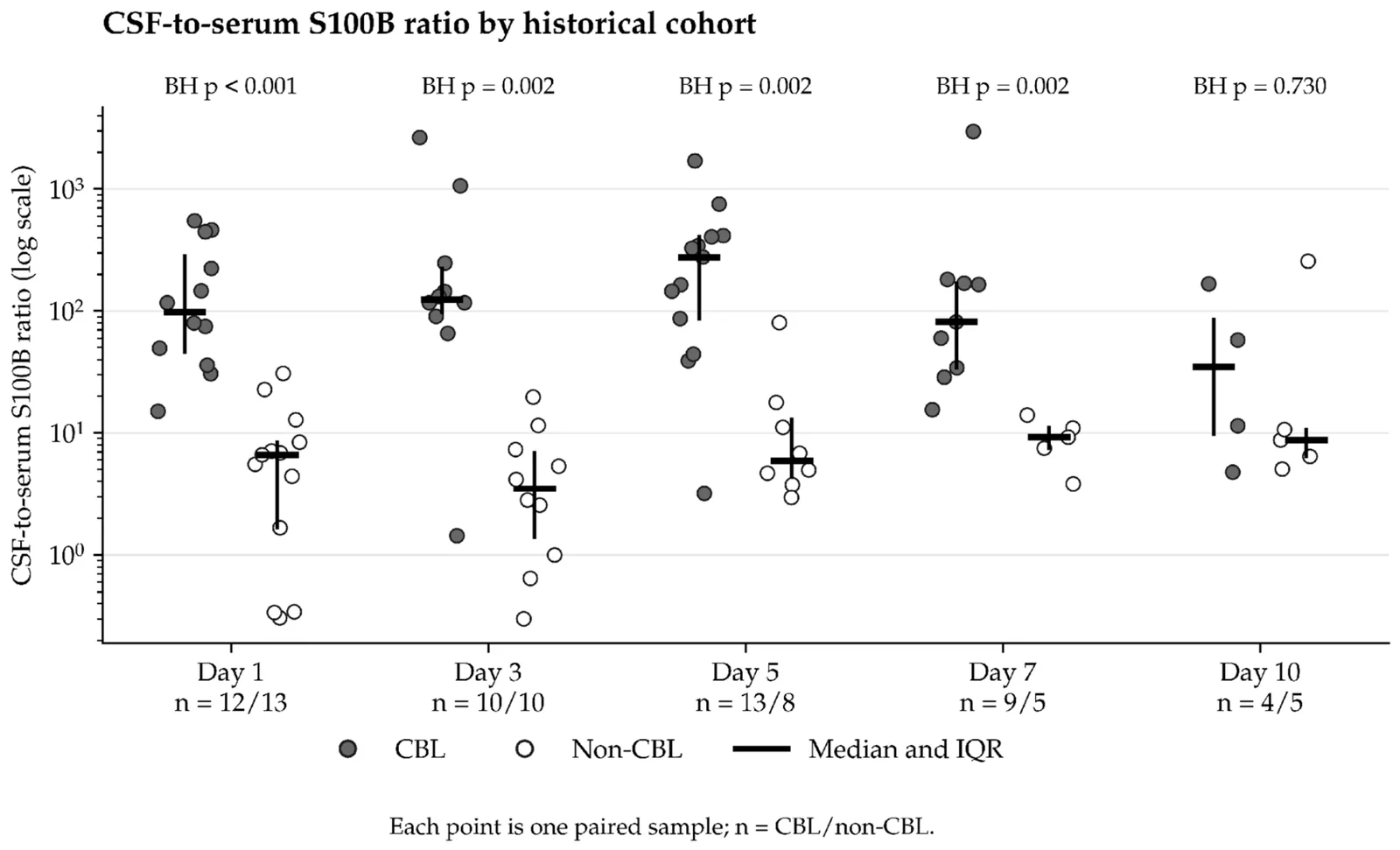
Individual CSF-to-serum S100B ratios from paired measurements obtained on the same sampling day. Filled circles indicate CBL and open circles non-CBL observations. Black horizontal marks show medians and vertical lines show IQRs. Horizontal displacement improves visibility without changing values. The vertical axis is logarithmic. Ratios include only pairs with strictly positive CSF and serum measurements; missing or zero measurements are excluded. Positive serum measurements below the lowest calibration standard are retained, which limits quantitative interpretation of high ratios. Sample sizes are CBL/non-CBL. Displayed BH-adjusted *p* values are taken from the accompanying ratio table, with correction across five days.

The CSF-to-serum S100B ratio was significantly higher in the CBL cohort on days 1, 3, 5, and 7, whereas no significant between-group difference was observed on day 10 (Table 5; Figure 3). Given the absence of significant differences in CSF S100B and the markedly lower serum concentrations in the CBL cohort, the higher ratio was primarily driven by the lower serum denominator. Accordingly, these ratio findings were considered exploratory.

### 3.4. Longitudinal Mixed-Effects Analysis of Serum and CSF S100B

In the primary additive longitudinal mixed-effects model, a significant overall between-cohort difference was observed for serum S100B (GMR 0.028, 95% CI 0.013–0.057; *p* < 0.001). The model including the CBL-by-day interaction showed no significant interaction (*p* = 0.218), providing no evidence that the between-cohort difference in serum S100B concentrations varied significantly over time. For CSF S100B, no significant overall between-cohort difference was observed (GMR 0.542, 95% CI 0.185–1.584; *p* = 0.263), and the CBL-by-day interaction was also non-significant (*p* = 0.305) (Table 6). The primary models accounted for repeated measurements and sampling day but did not adjust for admission disease severity or other potential confounders. Stratified sensitivity analyses should not be interpreted as a substitute for multivariable adjustment.

The sensitivity analysis restricted to days 3–10 confirmed the association observed for serum S100B (GMR 0.023, 95% CI 0.011–0.049; *p* < 0.001; CBL-by-day interaction *p* = 0.598), while CSF S100B remained non-significant (GMR 0.632, 95% CI 0.194–2.063; *p* = 0.447; CBL-by-day interaction *p* = 0.192) (Table 7). The serum association was also robust to alternative handling of values below the assay lower calibration boundary. After replacement of values below 0.0469 ng/mL by 0.0469 ng/mL or 0.02345 ng/mL, the corresponding GMRs were 0.071 (95% CI 0.042–0.122) and 0.048 (95% CI 0.027–0.085), respectively (both *p* < 0.001; Supplementary Table S4).

### 3.5. Sensitivity Analyses

Serum S100B remained significantly lower in the CBL cohort in analyses stratified by exclusive CSF drainage route: lumbar drainage (GMR 0.039, 95% CI 0.005–0.302; *p* = 0.002; CBL-by-day interaction *p* = 0.800) and external ventricular drainage (GMR 0.022, 95% CI 0.006–0.077; *p* < 0.001; CBL-by-day interaction *p* = 0.091). The formal CBL-by-drainage-route interaction for serum was not significant (*p* = 0.615). No significant overall between-cohort CSF S100B difference was identified in either drainage subgroup. A CBL-by-day interaction was observed for CSF within the lumbar-drainage subgroup (*p* = 0.009); however, this finding should be interpreted cautiously because of the very small non-CBL subgroup and sparse later CSF measurements.

In the endovascular coiling subgroup (*n* = 48; CBL *n* = 22, non-CBL *n* = 26), serum S100B remained significantly lower in the CBL cohort (GMR 0.033, 95% CI 0.014–0.081; *p* < 0.001), with no significant CBL-by-day interaction (*p* = 0.112). CSF S100B did not differ significantly between cohorts (GMR 0.497, 95% CI 0.120–2.058; *p* = 0.335; CBL-by-day interaction *p* = 0.577). Detailed analyses according to CSF drainage route and aneurysm-securing modality are presented in Supplementary Tables S1–S3.

### 3.6. Association of S100B Concentrations with Delayed Cerebral Ischemia

The incidence of DCI did not differ significantly between the CBL and non-CBL cohorts (15/26 vs. 20/41; OR 1.43, 95% CI 0.53–3.85; Fisher’s exact *p* = 0.617). In longitudinal mixed-effects analyses, serum S100B concentrations were higher in patients who developed DCI within the non-CBL cohort (GMR 2.75, 95% CI 1.35–5.61; *p* = 0.005). A similar direction was observed in the CBL cohort, although statistical significance was not reached (GMR 3.40, 95% CI 0.92–12.62; *p* = 0.067).

For CSF S100B, DCI was associated with higher concentrations in the CBL cohort (GMR 5.19, 95% CI 1.42–18.95; *p* = 0.013), whereas the corresponding association in the non-CBL cohort was not statistically significant (GMR 3.73, 95% CI 0.79–17.49; *p* = 0.095). The CBL-by-DCI interaction was not significant for either serum (*p* = 0.792) or CSF (*p* = 0.735), providing no evidence that the S100B–DCI relationship differed according to CBL exposure (Supplementary Table S5). To further characterize the temporal relationship between S100B concentrations and DCI, paired within-patient analyses compared the last available measurement before DCI onset with the first available measurement after onset; measurements obtained on the day of DCI onset were excluded. No significant paired change in serum S100B was observed in either cohort (CBL: *n* = 13, *p* = 0.685; non-CBL: *n* = 9, *p* = 0.910). Similarly, no significant paired change in CSF S100B was detected (CBL: *n* = 6, *p* = 0.688; non-CBL: *n* = 2, *p* = 1.000). All four Benjamini–Hochberg-adjusted *p* values were 1.000. Detailed results are presented in Supplementary Table S6 and Figure S1.

### 3.7. Serum and CSF S100B and Functional Outcomes

Unfavorable functional outcome was associated with higher serum S100B concentrations in both cohorts. At 3 months, the GMR for unfavorable versus favorable outcome was 6.99 (95% CI 2.05–23.79; *p* = 0.002) in the CBL cohort and 2.67 (95% CI 1.28–5.55; *p* = 0.009) in the non-CBL cohort. At hospital discharge, the corresponding GMRs were 9.20 (95% CI 2.57–32.95; *p* < 0.001) and 4.65 (95% CI 2.50–8.64; *p* < 0.001), respectively.

CSF S100B concentrations were associated with unfavorable functional outcome in the CBL cohort at 3 months (GMR 5.08, 95% CI 1.32–19.52; *p* = 0.018) and at hospital discharge (GMR 4.74, 95% CI 1.00–22.33; *p* = 0.049). The corresponding associations in the non-CBL cohort were not statistically significant. In-hospital mortality was associated with higher serum and CSF S100B concentrations in the non-CBL cohort (serum: GMR 4.01, 95% CI 1.85–8.66; CSF: GMR 8.85, 95% CI 2.65–29.53; both *p* < 0.001), whereas no statistically significant associations were observed in the CBL cohort. No significant CBL-by-outcome interactions were identified for either serum or CSF S100B (Supplementary Table S5). Exploratory correlations among S100B-derived indices are presented in Supplementary Figure S2.

## 4. Discussion

The principal finding of this study was a persistent association between CBL exposure and lower serum S100B concentrations from the earliest post-exposure measurement onward. In the CBL cohort, the first study sample was collected after treatment initiation, approximately 12 h after the first dose on average; therefore, no untreated S100B baseline measurement was available. In the primary longitudinal mixed-effects model, serum S100B concentrations were substantially lower in the CBL cohort (GMR 0.028, 95% CI 0.013–0.057; *p* < 0.001), while the CBL-by-day interaction was not significant (*p* = 0.218). The non-significant interaction provides no evidence that the between-cohort difference varied across sampling period, but it does not establish equivalent trajectories or stability of S100B concentrations over time.

The sensitivity analysis restricted to days 3–10 yielded a closely similar estimate (GMR 0.023, 95% CI 0.011–0.049; *p* < 0.001), indicating that the observed between-cohort difference was not dependent solely on the earliest post-exposure measurement. The association also persisted when alternative approaches were used to handle serum values below the assay calibration limit and in analyses stratified by CSF drainage route and aneurysm-securing modality (Supplementary Tables S1–S4). Nevertheless, the historical-cohort design does not permit attribution of this difference to CBL. Because treatment exposure was completely linked to the calendar period, the potential effects of CBL cannot be statistically separated from secular changes in clinical management, patient selection, laboratory procedures, or other era-related factors.

The clinical literature evaluating CBL after aSAH remains limited and heterogeneous. Woo et al. [44] did not demonstrate improvement in global functional performance at 6 months in a randomized pilot study, whereas Park et al. [45] reported a possible mortality benefit in a retrospective cohort, particularly among patients with severe hemorrhage. Kojder et al. [46] likewise suggested a possible mortality signal in a systematic review and meta-analysis. In the present cohort, however, CBL exposure was not associated with significant differences in DCI, TCD-detected vasospasm, in-hospital mortality, or functional outcome at discharge or at 3 months. Accordingly, the present findings do not provide clinical evidence of treatment efficacy.

S100B is an astroglial protein detectable in both CSF and peripheral blood after acute brain injury and has been widely investigated as a marker of astroglial and secondary brain damage [47,48,49,50,51,52]. At higher extracellular concentrations, S100B may participate in RAGE-dependent oxidative and neuroinflammatory signaling [53,54,55,56]. In aSAH, elevated or persistently increased serum S100B has been associated with greater injury severity and unfavorable outcome [57,58,59,60,61]. In the present study, higher longitudinal serum S100B concentrations were associated with unfavorable functional outcomes in both cohorts. These exploratory associations do not establish independent prognostic value because the outcome analyses were not adjusted for established prognostic factors and did not assess predictive performance or incremental value beyond clinical predictors.

The appearance of S100B in peripheral blood depends not only on intracranial release but also on transport across brain barriers and systemic clearance. Potential pathways include glymphatic and perivascular clearance, transport across the BBB and blood–CSF barrier, and arachnoid drainage [62]. These processes may be altered after aSAH as a consequence of neuroinflammation, endothelial dysfunction, BBB disruption, and impaired CSF circulation. Consequently, discordance between serum and CSF S100B concentrations should not automatically be interpreted as a direct indicator of altered astroglial injury.

CBL has been associated experimentally with neurotrophic-like, anti-inflammatory, antioxidant, and neuroprotective mechanisms [21,22,23,63,64,65]. Experimental endothelial studies have also suggested effects on vascular permeability and tight-junction proteins, including ZO-1, occludin, and claudin-5 [66,67]. These observations provide biological plausibility for investigating CBL in relation to circulating brain-injury biomarkers. However, the present data do not demonstrate that these mechanisms were responsible for the observed S100B pattern. Because the first available S100B measurement in the CBL cohort was obtained after treatment initiation and no untreated baseline was available, mechanistic explanations based on treatment-induced BBB stabilization or altered clearance cannot be inferred from this cohort.

The CSF findings further support cautious interpretation of the serum results. No significant overall between-cohort difference in CSF S100B was identified in the primary model (GMR 0.542, 95% CI 0.185–1.584; *p* = 0.263) or in the sensitivity analysis restricted to days 3–10 (GMR 0.632, 95% CI 0.194–2.063; *p* = 0.447). The CSF-to-serum ratio was substantially higher in the CBL cohort on days 1, 3, 5, and 7, but this finding was largely driven by the markedly lower serum denominator rather than by higher CSF S100B concentrations. The ratio should therefore be regarded as an exploratory measure and not as direct evidence that CBL altered S100B production, transport, or compartmental exchange.

Exploratory associations between S100B concentrations, DCI, and clinical outcomes were also observed, but their interpretation is limited by potential confounding by disease severity and uncertainty regarding temporal ordering.

DCI was associated with higher serum S100B concentrations in the non-CBL cohort, whereas the CBL-by-DCI interaction was not significant. The longitudinal analyses included measurements across the study period rather than being restricted to samples collected before DCI onset; therefore they cannot establish the predictive value of S100B for DCI. CSF associations were less consistent and were based on smaller samples.

The absence of statistically significant differences in DCI, vasospasm, mortality, or functional outcome is particularly important when interpreting the biomarker findings. A persistent between-cohort difference in circulating biomarker concentrations should not be considered surrogate evidence of therapeutic efficacy in the absence of corresponding clinical benefit, particularly when treatment exposure cannot be separated from the calendar period. The present findings therefore distinguish two separate observations: serum S100B concentrations differed markedly between the historical cohorts, whereas whether CBL itself modifies S100B biology after aSAH remains unresolved.

Future studies should use contemporaneous control groups or randomization, obtain rigorously standardized pre-treatment samples, and prespecify longitudinal models based on a true pre-treatment baseline. Parallel measurements of BBB permeability, endothelial injury, CSF turnover, and systemic S100B clearance could help determine whether changes in circulating S100B primarily reflect intracranial injury, barrier transport, or peripheral elimination. Such studies should also be adequately powered for clinical endpoints rather than relying on biomarker differences as surrogate measures of efficacy.

### Strengths and Limitations

A major strength of this study is the availability of serial serum S100B measurements across five predefined time points, together with serial CSF measurements in a clinically sampled subset, paired CSF-to-serum analyses, clinical outcomes, and prespecified sensitivity analyses. Importantly, the day-1 sample in the CBL cohort was explicitly recognized as a post-treatment measurement and was therefore not treated as a pre-treatment baseline.

The principal limitation is the retrospective, single-center, non-randomized historical-cohort design. CBL exposure was completely linked to the calendar period, preventing statistical separation of treatment exposure from secular changes in clinical management and other era-related factors. In addition, the first S100B measurement in the CBL cohort was obtained after treatment initiation, approximately 12 h after the first dose on average. Consequently, no untreated pre-exposure biomarker baseline was available.

These limitations preclude causal attribution of the lower serum S100B concentrations to CBL.

Second, the sample size was modest, particularly for CSF and paired CSF-to-serum analyses, and the number of available measurements decreased at later time points. Exclusion of a day in the sensitivity analysis further reduced the number of observations. These analyses should therefore be considered exploratory, particularly for CSF and ratio outcomes. A substantial proportion of serum measurement in the CBL cohort fell below the assay calibration range. Although alternative substitution approaches preserved the direction of the observed serum association, they produced different effect estimates and did not establish the quantitative accuracy of below-range measurements. Standardized sample processing and use of the same ELISA method do not exclude potential effects of storage duration, assay batch, or other analytical differences between recruitment periods. Moreover, sampling within a nominal study day may encompass biologically relevant variation, particularly in the absence of a pre-treatment measurement.

Third, CSF sampling was incomplete and was performed through both lumbar and ventricular drainage routes. Anatomical differences between lumbar and ventricular CSF compartments may contribute to biomarker variability. The paired analyses around DCI onset were limited by the small number of available within-patient pairs, particularly for CSF in the non-CBL cohort. These analyses were exploratory and assessed pre- versus post-DCI changes separately within each cohort rather than differences in change between cohorts. Accordingly, the absence of statistically significant paired changes should not be interpreted as evidence that DCI is unrelated to S100B concentrations.

Differences in treatment-era practices, including CSF diversion and aneurysm-securing modality, also remained potential sources of confounding despite the stratified sensitivity analyses.

Fourth, multiple secondary, subgroup, outcome, and correlation analyses were performed. Although BH correction was applied where appropriate, findings from small subgroups and secondary analyses may still reflect chance and should be considered hypothesis-generating.

Finally, no significant between-group differences were observed in DCI, TCD-detected vasospasm, mortality, or functional outcomes. The biomarker findings should therefore not be interpreted as surrogate evidence of clinical efficacy. The biological explanation for the observed persistent serum–CSF discordance remains unresolved and requires prospective investigation using contemporaneous controls, standardized laboratory procedures, direct assessment of BBB function, and evaluation of S100B transport and clearance. Given the limited sample size, the absence of statistically significant clinical outcome differences does not establish equivalence or exclude clinically relevant differences.

## 5. Conclusions

In this retrospective historical-cohort study of patients with aSAH, CBL exposure was associated with persistently lower serum S100B concentrations across days 1–10, including after exclusion of the earliest post-exposure measurement. No significant overall between-cohort difference was observed in CSF S100B concentrations. The CSF-to-serum S100B ratio was higher in the CBL cohort during the early and intermediate observation period, primarily reflecting the lower serum denominator. No statistically significant between-cohort differences were observed in DCI, TCD-detected vasospasm, mortality, or functional outcome. Because CBL exposure was completely linked to the treatment era and the first biomarker sample was obtained after treatment initiation, these exploratory associations cannot establish a direct pharmacological effect of CBL. Validation in larger, adequately powered prospective studies is required, particularly given the limited availability of CSF and paired measurements at later sampling points. Such studies should incorporate contemporaneous controls or randomization, standardized pre-treatment sampling, longitudinal assessment of serum and CSF S100B and their ratio, and clinically relevant endpoints to distinguish potential treatment effects from era-related confounding and determine the clinical relevance of the observed biomarker differences.

## Figures and Tables

**Table 1:** Comparison of aSAH Management Recommendations and the Institutional NICU Protocol Across Two Treatment Periods.

Management Domain	Guideline-Based Practice 2014–2017	Our NICU Protocol 2014–2017	Guideline-Based Practice 2023–2024	Our NICU Protocol 2023–2024	Main Change/Relevance for Cohort Comparison
Timing of aneurysm securing	Early aneurysm occlusion was recommended as soon as feasible.	Ruptured aneurysms were secured early, within 24 h of hospital admission, by clipping or coiling.	Aneurysm treatment is recommended as early as feasible, preferably within 24 h, with complete obliteration whenever possible.	Aneurysm securing remained an early priority and was performed within 24 h of admission.	Core strategy was similar in both periods; therefore, timing of aneurysm securing is unlikely to explain major between-era biomarker differences.
Aneurysm-securing modality	Both microsurgical clipping and endovascular coiling were accepted; selection depended on aneurysm anatomy, clinical status, and local expertise.	Both techniques were used. In the study cohort, coiling was less frequent than in the later period (63.4% coiling; 36.6% clipping).	Both modalities remain appropriate, with multidisciplinary selection and broad contemporary use of endovascular treatment when anatomically suitable.	Both techniques remained available. In the study cohort, coiling predominated (84.6% coiling; 15.4% clipping).	A shift toward endovascular treatment occurred over time and represents an era-related treatment difference; modality-specific sensitivity analyses are therefore relevant.
Nimodipine	Oral/enteral nimodipine was standard therapy to reduce the risk of poor neurological outcome related to DCI.	Oral or enteral nimodipine was routinely administered unless contraindicated.	Early enteral nimodipine remains a core Class I recommendation for aSAH management.	Oral or enteral nimodipine remained part of routine management.	No major protocol change; nimodipine was a common element of care in both periods.
Volume status and arterial pressure	Maintenance of euvolemia was recommended; prophylactic hypervolemia was not supported. Blood pressure management was individualized according to aneurysm status and clinical condition.	Euvolemia and adequate arterial blood pressure were maintained. Continuous invasive arterial blood pressure monitoring was routinely used, with individualized hemodynamic support.	Goal-directed euvolemia is emphasized; routine prophylactic hemodynamic augmentation and hypervolemia are avoided.	Euvolemia and adequate arterial pressure remained standard. Continuous invasive arterial blood pressure monitoring was routinely used, with individualized hemodynamic support.	The conceptual approach was similar, although contemporary care is more explicitly standardized around euvolemia and avoidance of prophylactic augmentation.
Treatment of symptomatic DCI	Induced hypertension was commonly used for symptomatic DCI after aneurysm securing; endovascular rescue therapy could be considered for refractory deterioration.	Clinically suspected or confirmed DCI was treated with induced hypertension while maintaining euvolemia, when not contraindicated. Balloon angioplasty and/or intra-arterial nimodipine were considered for persistent deterioration.	For symptomatic DCI, blood-pressure augmentation may be used when appropriate; endovascular rescue therapy is considered in selected refractory cases.	The same institutional strategy was maintained: induced hypertension with euvolemia as first-line therapy, with balloon angioplasty and/or intra-arterial nimodipine for insufficient response.	No fundamental change in the rescue strategy was documented in the manuscript.
Monitoring for cerebral vasospasm and DCI	Serial neurological examination and TCD were widely used; CTA/DSA and other imaging were obtained when clinically indicated.	Daily TCD ultrasonography was used to assess vasospasm; additional CT/CTA/MRI was performed when clinically indicated.	TCD remains useful, with CTA/CTP, EEG, and additional imaging used in selected patients, particularly when the clinical examination is limited.	Daily TCD remained routine; CT/CTA/MRI were obtained when clinically indicated.	Core.vasospasm/DCI surveillance was similar in both periods.
Multimodal neuromonitoring	Multimodal neuromonitoring was already established in neurocritical care, particularly in patients with severe acute brain injury or an unreliable neurological examination.	Multimodal neuromonitoring integrated serial clinical assessment, invasive arterial pressure monitoring, ICP/CPP monitoring when indicated, NIRS, and daily TCD.	Contemporary recommendations continue to support multimodal neuromonitoring in selected patients with high-grade aSAH or limited neurological examination.	The same multimodal strategy was maintained, integrating serial clinical assessment, invasive arterial pressure monitoring, ICP/CPP monitoring when indicated, NIRS, and daily TCD.	No major change; multimodal neuromonitoring was applied in both cohorts.
ICP and CPP monitoring	ICP monitoring was used in patients at risk of intracranial hypertension, with CPP interpreted together with ICP and systemic arterial pressure.	ICP and CPP were monitored when clinically indicated.	ICP/CPP monitoring remains an important component of neuromonitoring in high-grade aSAH when clinically indicated.	ICP and CPP continued to be monitored when clinically indicated.	No major change in the indication or use of ICP/CPP monitoring.
Near-infrared spectroscopy (NIRS)	Cerebral oxygenation monitoring could be incorporated as an adjunct within multimodal neurocritical care; NIRS was not a universal guideline requirement.	NIRS-based regional cerebral oxygenation monitoring was used as part of multimodal neuromonitoring.	NIRS remains an adjunctive non-invasive method within multimodal neuromonitoring rather than a universal guideline requirement.	NIRS-based regional cerebral oxygenation monitoring remained part of the institutional multimodal monitoring strategy.	No major protocol change; NIRS was used in both treatment periods.
Follow-up neuroimaging	Follow-up CT and vascular imaging were used after treatment and for neurological deterioration, according to clinical indication.	Follow-up CT was routinely obtained within 24 h after aneurysm securing; additional CT, CTA and/or MRI were performed as clinically indicated.	Repeat imaging remains integral to post-treatment assessment and investigation of new neurological deterioration or suspected DCI.	Follow-up CT within 24 h after aneurysm securing was maintained; additional CT/CTA/MRI remained clinically driven.	No major documented institutional change in the basic imaging strategy.
Acute hydrocephalus and EVD	EVD was the established first-line CSF diversion strategy for severe acute obstructive hydrocephalus.	EVD was preferred in severe acute hydrocephalus. In the study cohort, EVD was used in 29.3% of patients in the earlier treatment period.	Urgent CSF diversion is recommended for symptomatic hydrocephalus; EVD remains a standard option, with increasing emphasis on standardized drainage and weaning protocols.	EVD remained preferred for severe acute hydrocephalus. In the study cohort, EVD was used in 23.1% of patients in the later treatment period.	EVD remained a stable component of care; the major era-related CSF-diversion difference concerned lumbar drainage rather than ventricular drainage.
Lumbar CSF drainage	Evidence for routine lumbar drainage was limited; its role in preventing DCI or improving outcome was not firmly established.	LD was used selectively, particularly in communicating hydrocephalus or to facilitate clearance of subarachnoid blood. In the study cohort, LD was used in 12.2% of patients.	Contemporary evidence supports a broader role for lumbar CSF drainage in appropriately selected patients, including treatment of communicating hydrocephalus and facilitation of subarachnoid blood clearance.	LD was used more frequently in selected patients, particularly with communicating hydrocephalus or for blood-product clearance. In the study cohort, LD was used in 42.3% of patients.	This is one of the clearest treatment-era differences and a potential confounder for CSF biomarker analyses; drainage-route sensitivity analyses are important.
Cerebrolysin exposure	Cerebrolysin was not a guideline-recommended therapy for aSAH.	Cerebrolysin was not part of the institutional aSAH protocol in the earlier period.	Cerebrolysin is not specifically recommended in current aSAH guidelines.	Cerebrolysin 30 mL IV once daily was introduced as an institutional adjunctive treatment from day 1 after ictus in the later period.	CBL exposure was completely linked to treatment era; therefore, treatment exposure and calendar period cannot be separated statistically, and causal attribution is not possible.

Notes. The institutional protocol columns summarize management and observed treatment frequencies in the analyzed cohorts; these frequencies are not guideline recommendations. Guideline framework: AHA/ASA 2012, ESO 2013, AHA/ASA 2023, and neurocritical care recommendations. CBL exposure was completely linked to the treatment era. Abbreviations: aSAH, aneurysmal subarachnoid hemorrhage; CBL, Cerebrolysin; CPP, cerebral perfusion pressure; CSF, cerebrospinal fluid; CT, computed tomography; CTA, CT angiography; CTP, CT perfusion; DCI, delayed cerebral ischemia; DSA, digital subtraction angiography; EEG, electroencephalography; EVD, external ventricular drainage; ICP, intracranial pressure; LD, lumbar drainage; MRI, magnetic resonance imaging; NICU, neurointensive care unit; NIRS, near-infrared spectroscopy; TCD, transcranial Doppler. Guideline references: [28,29,30,31].

**Table 3:** Serum S100B Concentrations According to CBL Exposure.

Day	CBL, Median (IQR), *n*	Non-CBL, Median (IQR), *n*	*p* Value	BH-Adjusted *p*
Day 1	0.0438 (0.0219–0.3289), *n* = 25	1.771 (0.600–3.433), *n* = 35	<0.001	<0.001
Day 3	0.0467 (0.0039–0.2190), *n* = 21	0.930 (0.613–3.682), *n* = 35	<0.001	<0.001
Day 5	0.0441 (0.0064–0.1170), *n* = 23	0.900 (0.643–4.489), *n* = 36	<0.001	<0.001
Day 7	0.0282 (0.0132–0.0545), *n* = 18	0.700 (0.192–3.520), *n* = 30	<0.001	<0.001
Day 10	0.0502 (0.0230–0.1149), *n* = 13	0.930 (0.700–3.945), *n* = 18	<0.001	<0.001

Data are presented as median (IQR); *n* denotes the number of available measurements at each time point. Between-group comparisons were performed using the two-sided Mann–Whitney U test. *p* values were adjusted across the five time points using the Benjamini–Hochberg (BH) procedure. In the CBL cohort, day 1 represents an early post-exposure measurement.

**Table 4:** Cerebrospinal Fluid S100B Concentrations According to CBL Exposure.

Day	CBL, Median (IQR), *n*	Non-CBL, Median (IQR), *n*	*p* Value	BH-Adjusted *p*
Day 1	4.237 (2.516–29.629), *n* = 12	18.085 (1.660–24.702), *n* = 14	0.898	0.898
Day 3	4.532 (2.285–19.207), *n* = 11	7.381 (0.650–21.397), *n* = 11	0.844	0.898
Day 5	9.827 (1.622–29.745), *n* = 16	16.030 (9.906–25.614), *n* = 9	0.591	0.898
Day 7	3.489 (1.501–3.762), *n* = 10	11.028 (1.950–42.513), *n* = 6	0.368	0.898
Day 10	2.765 (1.671–3.925), *n* = 4	20.284 (15.370–23.931), *n* = 5	0.190	0.898

Data are presented as median (IQR); *n* denotes the number of available measurements at each time point. Between-group comparisons were performed using the two-sided Mann–Whitney U test. *p* values were adjusted across the five time points using the Benjamini–Hochberg (BH) procedure.

**Table 5:** CSF-to-Serum S100B Ratio According to CBL Exposure.

Day	CBL, Median (IQR), *n*	Non-CBL, Median (IQR), *n*	*p* Value	BH-Adjusted *p*
Day 1	98.50 (46.19–279.87), *n* = 12	6.62 (1.67–8.41), *n* = 13	<0.001	<0.001
Day 3	124.27 (97.20–222.30), *n* = 10	3.50 (1.39–6.83), *n* = 10	0.001	0.002
Day 5	277.85 (86.90–405.76), *n* = 13	5.90 (4.45–12.79), *n* = 8	<0.001	0.002
Day 7	80.98 (34.21–168.74), *n* = 9	9.24 (7.50–11.01), *n* = 5	<0.001	0.002
Day 10	34.58 (9.79–85.04), *n* = 4	8.75 (6.41–10.64), *n* = 5	0.730	0.730

The ratio was calculated from paired CSF and serum measurements obtained on the same study day. Data are presented as median (IQR). Between-group comparisons were performed using the two-sided Mann–Whitney U test with Benjamini–Hochberg adjustment across the five time points. Measurements with serum S100B = 0 were excluded because the ratio is undefined. The ratio was considered exploratory because it is highly sensitive to low serum S100B concentrations.

**Table 6:** Primary Longitudinal Mixed-Effects Models for Serum and CSF S100B (Days 1, 3, 5, 7, and 10).

Compartment	Measurements/Patients	β (CBL)	GMR (CBL/Non-CBL)	95% CI	*p* Value (CBL)	CBL × Day *p* Value	Time Effect *p* Value
Serum S100B	254/67 (CBL 100/26; non-CBL 154/41)	−3.590	0.028	0.013–0.057	<0.001	0.218	0.049
CSF S100B	98/36 (CBL 53/20; non-CBL 45/16)	−0.613	0.542	0.185–1.584	0.263	0.305	0.072

Linear mixed-effects models included a patient-specific random intercept. S100B concentrations were transformed as ln(S100B + c), with c = 0.0008 ng/mL for serum and c = 0.03 ng/mL for CSF. Fixed effects included CBL exposure, categorical study day, and the CBL × day interaction. Because the interaction was non-significant, the overall CBL effect was estimated from an additive model and is reported as a geometric mean ratio (GMR; CBL/non-CBL) with 95% CI. Day 1 represents an early post-exposure measurement in the CBL cohort.

**Table 7:** Sensitivity Mixed-Effects Analysis Restricted to Days 3, 5, 7, and 10.

Compartment	Measurements/Patients	β (CBL)	GMR (CBL/Non-CBL)	95% CI	*p* Value (CBL)	CBL × Day *p* Value	Time Effect *p* Value
Serum S100B	194/66 (CBL 75/25; non-CBL 119/41)	−3.787	0.023	0.011–0.049	<0.001	0.598	0.485
CSF S100B	72/31 (CBL 41/19; non-CBL 31/12)	−0.459	0.632	0.194–2.063	0.447	0.192	0.150

The sensitivity analysis excluded day-1 measurements and did not adjust for day-1 S100B because the first measurement in the CBL cohort was obtained after treatment initiation. Model specification and transformation were otherwise identical to those used in the primary analysis. GMR values below 1 indicate lower S100B concentrations in the CBL cohort. CBL, Cerebrolysin; CI, confidence interval; CSF, cerebrospinal fluid; GMR, geometric mean ratio.

## Data Availability

Anonymized data may be made available from the corresponding author upon reasonable request.

## Supplementary Materials

The following supporting information can be downloaded at https://www.mdpi.com/article/10.3390/jcm15218734/s1: Table S1, Longitudinal Mixed-Effects Sensitivity Analyses According to CSF Drainage Route and Aneurysm-Securing Modality; Tables S2, Time-Specific Serum and CSF S100B Comparisons According to Exclusive CSF Drainage Route; Table S3, Time-Specific S100B Comparisons in Patients Treated with Endovascular Coiling.Time-Specific S100B Comparisons in Patients Treated with Endovascular Coiling; Table S4, Sensitivity Analyses for Serum S100B Measurements Below the Assay Lower Calibration Boundary (0.0469 ng/mL); Table S5, Associations of Longitudinal Serum and CSF S100B with DCI and Clinical Outcomes Within CBL and non-CBL Cohorts; Table S6, Paired S100B concentrations before and after DCI onset, stratified by Cerebrolysin exposure; Table S7, Procedures and longitudinal models; Figure S1, Paired serum (A) and CSF (B) S100B concentrations before and after DCI onset, stratified by Cerebrolysin exposure; Figure S2, Spearman Correlation Matrices of Patient-Level S100B-Derived Indices During the Study Period.

## Abbreviations

The following abbreviations are used in this manuscript:

APACHE II	Acute Physiology and Chronic Health Evaluation II	GMR	geometric mean ratio
aSAH	aneurysmal subarachnoid hemorrhage	ICH	intracerebral hematoma
BBB	blood–brain barrier	ICP	intracranial pressure
BH	Benjamini–Hochberg	ICU	intensive care unit
BMI	body mass index	IQR	interquartile range
CBL	Cerebrolysin	LD	lumbar drainage
CI	confidence interval	m-GCS	motor Glasgow Coma Scale
CPP	cerebral perfusion pressure	mFS	modified Fisher scale
CSF	cerebrospinal fluid	MRI	magnetic resonance imaging
CT	computed tomography	mRS	modified Rankin Scale
CTA	computed tomography angiography	NIRS	near-infrared spectroscopy
DCI	delayed cerebral ischemia	OR	odds ratio
DSA	digital subtraction angiography	rpm	Revolutions per minute
EEG	electroencephalography	S100B	S100 calcium-binding protein B
ELISA	enzyme-linked immunosorbent assay	SD	standard deviation
EVD	external ventricular drainage	SEBES	Subarachnoid Hemorrhage Early Brain Edema Score
FOUR	Full Outline of UnResponsiveness	TCD	transcranial Doppler
GCS	Glasgow Coma Scale	WFNS	World Federation of Neurosurgical Societies
