# Supplementary material for: Longitudinal Serum and Cerebrospinal Fluid S100B Concentrations After Aneurysmal Subarachnoid Hemorrhage: An Exploratory Comparison of Historical Cohorts With and Without Cerebrolysin Exposure
Source: J Clin Med. 2026 Nov 9;15(21):8734. doi: 10.3390/jcm15218734 (PMC13763813; doi:10.3390/jcm15218734)
Supplement: Supplementary file 1 [file jcm-15-08734-s001.zip › jcm-4592368-supplementary.pdf]

Supplementary Materials

Longitudinal Serum and Cerebrospinal Fluid S100B Concentrations After Aneurysmal Subarachnoid Hemorrhage: An Exploratory Comparison of Historical Cohorts With and Without Cerebrolysin Exposure

Małgorzata Burzyńska, Adrianna Lebiedzińska, Jarosław Kędziora, Barbara Dragan, Bogusława Lechowicz-Głogowska, Barbara Adamik, Marek Wełna, Waldemar Goździk

Supplementary Methods

S1. Sensitivity Analyses for the Serum Assay Lower Calibration Boundary

The manufacturer-specified lower calibration boundary of the S100B assay was 0.0469 ng/mL. In the primary exploratory analysis, recorded values below this boundary were retained as measured values. To assess robustness, serum values below 0.0469 ng/mL were alternatively replaced by either 0.0469 ng/mL or 0.02345 ng/mL (half the lower calibration boundary). Substituted concentrations were natural-log transformed and analyzed using the same longitudinal random-intercept mixed-effects structure with CBL exposure, categorical study day, and the CBL-by-day interaction. When the interaction was not significant, an additive model was used to estimate the overall CBL/non-CBL geometric mean ratio. These analyses were considered sensitivity analyses rather than formal assay validation.

S2. Exploratory Spearman Correlation Analysis

Patient-level S100B-derived indices were calculated for serum, CSF, and the CSF-to-serum S100B ratio across study days 1, 3, 5, 7, and 10. Day-1, peak, and median values were evaluated. Peak and median indices required at least two serial measurements. Ratio-derived indices were calculated only from same-day paired CSF and serum measurements with serum S100B > 0. Spearman rank correlations were calculated for the overall cohort and separately for the CBL and non-CBL cohorts using pairwise complete observations. P values for unique pairwise correlations within each matrix were adjusted using the Benjamini–Hochberg procedure. Because several indices were mathematically related and the ratio contains serum S100B in its denominator, these analyses were considered exploratory.

Table S1. Longitudinal Mixed-Effects Sensitivity Analyses According to CSF Drainage Route and Aneurysm-Securing Modality.

Stratum	Compartment	Measurements / patients	GMR CBL/non-CBL	95% CI	*p* (CBL)	CBL × day *p*
Lumbar drainage (exclusive)	Serum	60 / 15 (CBL 11; non-CBL 4)	0.039	0.005–0.302	0.002	0.800
Lumbar drainage (exclusive)	CSF	41 / 15 (CBL 11; non-CBL 4)	0.942	0.145–6.132	0.950	0.009
External ventricular drainage (exclusive)	Serum	65 / 17 (CBL 6; non-CBL 11)	0.022	0.006–0.077	<0.001	0.091
External ventricular drainage (exclusive)	CSF	46 / 14 (CBL 5; non-CBL 9)	0.750	0.159–3.544	0.717	0.349
Endovascular coiling	Serum	183 / 48 (CBL 22; non-CBL 26)	0.033	0.014–0.081	<0.001	0.112
Endovascular coiling	CSF	68 / 25 (CBL 17; non-CBL 8)	0.497	0.120–2.058	0.335	0.577
Surgical clipping	Serum	71 / 19 (CBL 4; non-CBL 15)	0.018	0.005–0.070	<0.001	0.512
Surgical clipping	CSF	30 / 11 (CBL 3; non-CBL 8)	0.581	0.056–5.993	0.648	0.851

Models used patient-specific random intercepts and ln(S100B + c), with c = 0.0008 ng/mL for serum and 0.03 ng/mL for CSF. The GMR is from the additive model; when the CBL-by-day interaction is significant, the additive GMR should be interpreted cautiously. Formal CBL-by-drainage-route interaction p values were 0.615 for serum and 0.760 for CSF; formal CBL-by-aneurysm-securing-modality interaction p values were 0.518 for serum and 0.772 for CSF. The clipping subgroup included only four CBL-exposed patients overall, and CSF data were available for only three of them.

Table S2. Time-Specific Serum and CSF S100B Comparisons According to Exclusive CSF Drainage Route.

Route	Compartment	Day	CBL, median (IQR), *n*	non-CBL, median (IQR), *n*	*p*	BH-adjusted *p*
LD	Serum	Day 1	0.109 (0.052–0.597), *n* = 10	2.325 (1.508–4.639), *n* = 4	0.008	0.027
LD	Serum	Day 3	0.138 (0.047–0.347), *n* = 9	1.540 (0.635–5.913), *n* = 4	0.053	0.089
LD	Serum	Day 5	0.049 (0.025–0.104), *n* = 11	1.450 (0.500–5.051), *n* = 4	0.011	0.027
LD	Serum	Day 7	0.038 (0.016–0.079), *n* = 7	0.390 (0.075–2.300), *n* = 4	0.073	0.091
LD	Serum	Day 10	0.070 (0.023–0.115), *n* = 5	2.544 (1.302–3.787), *n* = 2	0.571	0.571
LD	CSF	Day 1	18.698 (2.810–33.945), *n* = 8	9.325 (0.818–26.990), *n* = 4	0.683	0.854
LD	CSF	Day 3	18.567 (5.661–28.609), *n* = 6	34.956 (17.508–52.404), *n* = 2	1.000	1.000
LD	CSF	Day 5	21.690 (4.217–33.504), *n* = 9	39.148 (27.589–50.707), *n* = 2	0.436	0.854
LD	CSF	Day 7	3.734 (1.736–15.260), *n* = 6	78.127 (78.127–78.127), *n* = 1	0.286	0.854
LD	CSF	Day 10	2.765 (2.223–3.308), *n* = 2	15.370 (15.370–15.370), *n* = 1	0.667	0.854
EVD	Serum	Day 1	0.021 (0.020–0.204), *n* = 6	1.771 (0.600–2.753), *n* = 11	0.024	0.029
EVD	Serum	Day 3	0.009 (0.004–0.020), *n* = 5	1.750 (0.930–3.910), *n* = 9	0.011	0.019
EVD	Serum	Day 5	0.014 (0.008–0.044), *n* = 5	3.155 (0.746–5.277), *n* = 8	0.010	0.019
EVD	Serum	Day 7	0.034 (0.021–0.037), *n* = 5	1.631 (0.348–1.911), *n* = 8	0.004	0.019
EVD	Serum	Day 10	0.028 (0.014–0.065), *n* = 3	0.700 (0.613–3.650), *n* = 5	0.071	0.071
EVD	CSF	Day 1	3.954 (3.158–4.237), *n* = 3	18.410 (2.285–21.266), *n* = 7	0.493	1.000
EVD	CSF	Day 3	2.285 (1.991–4.354), *n* = 4	5.941 (0.675–17.480), *n* = 8	0.799	1.000
EVD	CSF	Day 5	13.337 (6.317–14.443), *n* = 5	11.658 (2.927–16.041), *n* = 6	0.927	1.000
EVD	CSF	Day 7	3.083 (2.135–3.459), *n* = 4	5.698 (0.700–16.357), *n* = 5	0.413	1.000
EVD	CSF	Day 10	1.643 (1.643–1.643), *n* = 1	20.284 (10.492–29.552), *n* = 3	1.000	1.000

Only patients managed exclusively with LD or EVD were included. One patient who underwent both procedures was excluded from route-specific analyses. P values are from two-sided Mann–Whitney U tests; BH adjustment was applied across the five time points within each route and compartment.

Table S3. Time-Specific S100B Comparisons in Patients Treated with Endovascular Coiling.

Compartment	Day	CBL, median (IQR), *n*	non-CBL, median (IQR), *n*	*p*	BH-adjusted *p*
Serum	Day 1	0.044 (0.023–0.329), *n* = 21	0.700 (0.600–2.898), *n* = 22	<0.001	<0.001
Serum	Day 3	0.062 (0.003–0.199), *n* = 18	0.700 (0.600–2.397), *n* = 22	<0.001	<0.001
Serum	Day 5	0.045 (0.006–0.123), *n* = 19	0.718 (0.662–3.579), *n* = 22	<0.001	<0.001
Serum	Day 7	0.022 (0.011–0.057), *n* = 16	0.700 (0.336–3.390), *n* = 19	<0.001	<0.001
Serum	Day 10	0.070 (0.030–0.131), *n* = 11	0.930 (0.700–3.735), *n* = 13	0.003	0.003
CSF	Day 1	6.567 (2.751–32.456), *n* = 10	14.768 (5.220–24.352), *n* = 6	0.958	0.958
CSF	Day 3	9.050 (4.235–28.084), *n* = 9	7.381 (2.600–21.397), *n* = 7	0.758	0.947
CSF	Day 5	6.317 (1.411–33.469), *n* = 13	16.918 (9.906–25.614), *n* = 5	0.703	0.947
CSF	Day 7	3.549 (1.089–3.790), *n* = 9	16.357 (11.027–33.794), *n* = 3	0.100	0.250
CSF	Day 10	3.850 (2.765–4.000), *n* = 3	23.931 (22.108–31.376), *n* = 3	0.100	0.250

The coiling subgroup comprised 48 patients (CBL *n* = 22; non-CBL *n* = 26). P values are from two-sided Mann–Whitney U tests with BH adjustment across five time points within each compartment.

Table S4. Sensitivity Analyses for Serum S100B Measurements Below the Assay Lower Calibration Boundary (0.0469 ng/mL).

Panel A. Longitudinal mixed-effects sensitivity models.

Strategy	Replacement value	Measurements / patients	GMR CBL/non-CBL	95% CI	*p* (CBL)	CBL × day *p*
Replacement at lower calibration boundary	0.04690 ng/mL	254 / 67	0.071	0.042–0.122	<0.001	0.600
Replacement at half the lower calibration boundary	0.02345 ng/mL	254 / 67	0.048	0.027–0.085	<0.001	0.496

Panel B. Frequency of serum measurements below 0.0469 ng/mL.

Group	Day	Available serum *n*	Below boundary *n*	Below boundary (%)
CBL	Day 1	25	13	52.0%
CBL	Day 3	21	11	52.4%
CBL	Day 5	23	13	56.5%
CBL	Day 7	18	13	72.2%
CBL	Day 10	13	6	46.2%
non-CBL	Day 1	35	0	0.0%
non-CBL	Day 3	35	0	0.0%
non-CBL	Day 5	36	0	0.0%
non-CBL	Day 7	30	0	0.0%
non-CBL	Day 10	18	0	0.0%

Values below the calibration boundary occurred only in the CBL cohort in this dataset. These robustness analyses support persistence of the between-cohort serum difference under conservative substitution assumptions but do not address calendar-period confounding or establish a causal pharmacological effect.

Table S5. Associations of Longitudinal Serum and CSF S100B with DCI and Clinical Outcomes Within CBL and non-CBL Cohorts.

Outcome	Compartment	CBL: GMR (95% CI); *p*	non-CBL: GMR (95% CI); *p*	CBL × outcome *p*
Delayed cerebral ischemia (yes vs no)	Serum	3.40 (0.92–12.62); 0.067	2.75 (1.35–5.61); 0.005	0.792
Delayed cerebral ischemia (yes vs no)	CSF	5.19 (1.42–18.95); 0.013	3.73 (0.79–17.49); 0.095	0.735
Unfavorable mRS at 3 months (3–6 vs 0–2)	Serum	6.99 (2.05–23.79); 0.002	2.67 (1.28–5.55); 0.009	0.159
Unfavorable mRS at 3 months (3–6 vs 0–2)	CSF	5.08 (1.32–19.52); 0.018	1.98 (0.34–11.62); 0.449	0.418
Unfavorable mRS at discharge (3–6 vs 0–2)	Serum	9.20 (2.57–32.95); <0.001	4.65 (2.50–8.64); <0.001	0.295
Unfavorable mRS at discharge (3–6 vs 0–2)	CSF	4.74 (1.00–22.33); 0.049	1.98 (0.34–11.62); 0.449	0.453
In-hospital mortality (death vs survival)	Serum	1.83 (0.37–9.15); 0.461	4.01 (1.85–8.66); <0.001	0.337
In-hospital mortality (death vs survival)	CSF	1.86 (0.38–9.02); 0.440	8.85 (2.65–29.53); <0.001	0.120

GMRs compare the outcome category listed first with the reference category listed second (e.g., DCI vs no DCI; unfavorable vs favorable outcome; death vs survival). Models included categorical study day and patient-specific random intercepts. Interaction p values were obtained from pooled models testing whether the S100B–outcome association differed according to CBL exposure.

To further characterize temporal relationship between S100B concentrations and DCI, we performed an additional paired analyses comparing the last available measurement before DCI onset with the first available measurement after onset in the same patient. Measurements obtained on the day of DCI onset were excluded. No significant paired change in serum S100B was observed in either cohort (CBL: n = 13, p = 0.685; non-CBL: n = 9, p = 0.910). Similarly, no significant paired change in CSF S100B was observed (CBL: n = 6, p = 0.688; non-CBL: n = 2, p = 1.000). All four Benjamini–Hochberg-adjusted p values were 1.000. These results are presented in Supplementary Table S1 and Figure S1. The small number of CSF pairs, particularly in the non-CBL cohort, limits interpretation. These within-cohort comparisons do not test whether changes differ between cohorts.

These results are presented in Supplementary Table S1 and Figure S1. We have clarified that these analyses are exploratory and limited by the small number of paired observations, particularly for CSF in the non-CBL cohort. Furthermore, the within-cohort comparisons do not directly test whether changes associated with DCI differ between the two cohorts.

Accordingly, we do not interpret the absence of statistically significant paired changes as evidence that DCI has no effect on S100B concentrations. Rather, the available data do not demonstrate a significant within-patient change across DCI onset in these exploratory analyses

Table S6 Paired S100B concentrations before and after DCI onset, stratified by Cerebrolysin exposure.

Sample	Group	Pairs, *n*	Before DCI (ng/mL)	After DCI (ng/mL)	*p*	BH-adjusted, *p*
Serum	Non-CBL	9	4.340 (0.700–6.820)	4.960 (3.735–6.408)	0.910	1.000
Serum	CBL	13	0.049 (0.034–0.190)	0.112 (0.052–0.147)	0.685	1.000
CSF	Non-CBL	2	43.940 (34.777–53.103)	51.029 (37.480–64.578)	1.000	1.000
CSF	CBL	6	16.151 (2.710–31.800)	3.764 (3.581–26.767)	0.688	1.000

Values are median (IQR), ng/mL. The last available measurement before DCI onset was compared with the first available measurement after onset in the same patient. Measurements obtained on the day of DCI onset were excluded. Two-sided Wilcoxon signed-rank tests were performed separately within each cohort and sample type; Benjamini–Hochberg correction was applied across the four comparisons. Only two CSF pairs were available in the non-CBL cohort, limiting interpretation. These tests do not assess whether changes differ between cohorts. CBL, Cerebrolysin; DCI, delayed cerebral ischemia; CSF, cerebrospinal fluid; IQR, interquartile range; BH, Benjamini–Hochberg.

Stratified paired analyses showed no significant change in serum S100B concentrations between the last available measurement before DCI onset and the first available measurement after DCI onset in either Cerebrolysin-exposed patients (n = 13; p = 0.685) or patients without Cerebrolysin exposure (n = 9; p = 0.910). Similarly, no significant paired change was observed in CSF S100B concentrations in either group (CBL: n = 6, p = 0.688; non-CBL: n = 2, p = 1.000). All four comparisons remained non-significant after Benjamini–Hochberg correction (adjusted p = 1.000 for each comparison). Interpretation of the CSF analysis in the non-CBL group is limited by the availability of only two paired observations.

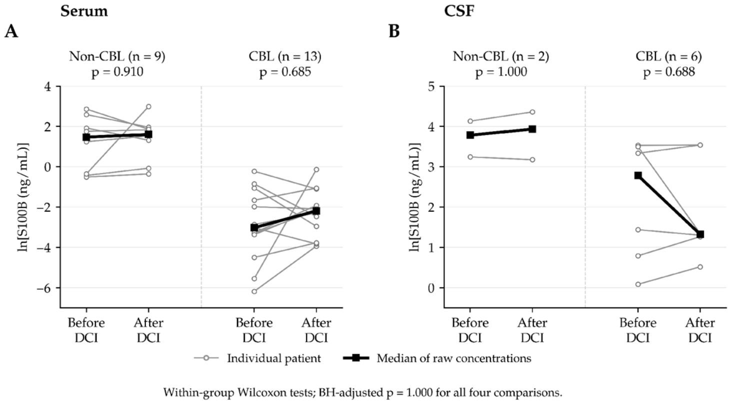

Figure S1. Paired serum (A) and CSF (B) S100B concentrations before and after DCI onset, stratified by Cerebrolysin exposure. Thin gray lines connect the last pre-DCI and first post-DCI measurements in each patient. Thick black lines connect medians of raw concentrations, plotted after natural-log transformation. Cohorts share a y-axis within each panel. All included pairs were positive; day-of-onset measurements were excluded. Displayed p values are from two-sided within-cohort Wilcoxon signed-rank tests; all four BH-adjusted p values equal 1.000. These tests do not compare changes between cohorts. CBL, Cerebrolysin; DCI, delayed cerebral ischemia; CSF, cerebrospinal fluid; BH, Benjamini–Hochberg.

S.7 SMDs and the severity-adjusted mixed-effects models.

Table S7. Procedures and longitudinal models

**Procedure**	**CBL, n = 26**	**Non-CBL, n = 41**	**Absolute SMD**
Endovascular treatment	22 (84.6%)	26 (63.4%)	0.498
Surgical clipping	4 (15.4%)	15 (36.6%)	0.498
Surgery for intracerebral hemorrhage	6 (23.1%)	6 (14.6%)	0.217
Decompressive craniectomy	6 (23.1%)	4 (9.8%)	0.366
Lumbar drainage	11 (42.3%)	5 (12.2%)	0.719
External ventricular drainage	6 (23.1%)	12 (29.3%)	0.141

**Sample**	**Adjustment**	**Patients / values**	**CBL / non-CBL ratio (95% CI)**	**p**
Serum	APACHE II and WFNS	65 / 244	0.030 (0.017 to 0.051)	<0.001
Serum	Plus LD EVD and securing	65 / 244	0.030 (0.016 to 0.056)	<0.001
CSF	APACHE II and WFNS	35 / 97	0.623 (0.257 to 1.673)	0.317
CSF	Plus LD EVD and securing	35 / 97	0.627 (0.198 to 1.983)	0.376

All models include categorical day and a patient random intercept. Primary CIs are patient-bootstrap percentile intervals; extended-model CIs are asymptotic Wald intervals. Primary p values are Holm-adjusted across serum and CSF; extended-model p values are exploratory ML likelihood-ratio tests. Recorded positive values are used here. Zero-only serum records exclude two CBL patients; one CSF patient has only a zero measurement.

Sensitivity to the lowest calibration standard

**Handling below 0.0469 ng/mL**	**Serum ratio (95% Wald CI)**	**CSF ratio (95% Wald CI)**
Replace by 0.0469	0.056 (0.034 to 0.092)	0.543 (0.186 to 1.589)
Replace by 0.0469 / sqrt(2)	0.045 (0.027 to 0.075)	0.537 (0.181 to 1.597)

In CBL patients, 56/100 serum values lie below the lowest calibration standard. Substitution includes all 67 serum patients and all 36 CSF patients. It is a sensitivity assumption, not a validated quantification method. Many concentrations also exceed the highest standard of 3 ng/mL; documented sample dilution and back-calculation are needed to assess those values

Methods

Baseline characteristics were assessed using standardized mean differences (SMDs), calculated as the difference between group means divided by the square root of the average group variances. For binary variables, the denominator used the average of p(1-p) in the two groups. Absolute SMDs were reported without using significance tests to establish balance. An absolute SMD of 0.10 was used as a descriptive reference, not a test of clinical importance. Ordinal scores were summarized by medians and IQRs, with SMDs calculated from their means and SDs [1]. Separate Gaussian linear mixed models were fitted to natural-log-transformed serum and CSF S100B, with a patient-specific random intercept, CBL group, categorical sampling day, admission APACHE II, and admission WFNS grade. APACHE II and WFNS were retained on clinical grounds, without selection by baseline p values. Each score was entered as a one-point linear term. Fixed-effect and variance estimates used restricted maximum likelihood. The CBL association was tested

by a one-degree-of-freedom likelihood-ratio test using maximum-likelihood fits, with Holm correction across the two compartment-specific tests. Confidence intervals used 1500 nonparametric bootstrap resamples of patients within CBL groups, retaining each selected patient's complete measurement series. The primary exploratory models used recorded positive values; zero and missing measurements were excluded, without imputing missing observations. Sensitivity models additionally included LD and EVD, followed by endovascular securing, or age and hypertension. Separate sensitivity analyses replaced measurements below the lowest calibration standard (0.0469 ng/mL) by that standard or by the standard divided by the square root of two. These substitutions are assumptions and do not validate measurements outside the assay range. Interaction between group and categorical day was assessed separately by a four-degree-of-freedom likelihood-ratio test.

Results

We assessed between-group differences using absolute standardized mean differences (SMDs), which indicated imbalances in lumbar drainage (0.719), diabetes mellitus (0.511), endovascular treatment (0.498), APACHE II (0.497), hypertension (0.459), age (0.393), and decompressive craniectomy (0.366). Baseline characteristics and treatment procedures were therefore presented separately.

To account for differences in initial disease severity, separate longitudinal mixed-effects models were fitted for serum and CSF S100B, including CBL group, categorical sampling day, admission APACHE II, admission WFNS grade, and a patient-specific random intercept. Covariates were selected on clinical grounds rather than baseline p values.

The serum association remained significant after severity adjustment [geometric mean ratio 0.030 (95% CI 0.017–0.051); adjusted $p < 0.001$] and in sensitivity models additionally accounting for drainage and aneurysm-securing method. No significant overall association was found for CSF [ratio 0.623 (95% CI 0.257–1.673); adjusted $p = 0.317$].

Diabetes was too infrequent for reliable adjustment, and decompressive craniectomy was not included as a baseline covariate because its timing relative to admission was uncertain. Importantly, adjustment for measured severity cannot eliminate residual confounding between historical cohorts, and the high proportion of serum values below the lowest calibration standard limits precision of the effect-size estimate. These findings therefore represent adjusted associations rather than a causal effect of Cerebrolysin.

All models converged successfully. Serum residuals were approximately symmetric, whereas CSF residuals showed heavier tails, supporting cautious interpretation of the smaller CSF dataset. APACHE II and WFNS were moderately correlated ($r = 0.628$). Group-by-day interactions were not significant for serum ($p = 0.345$) or CSF ($p = 0.200$).

Figure S2. Spearman Correlation Matrices of Patient-Level S100B-Derived Indices During the Study Period.

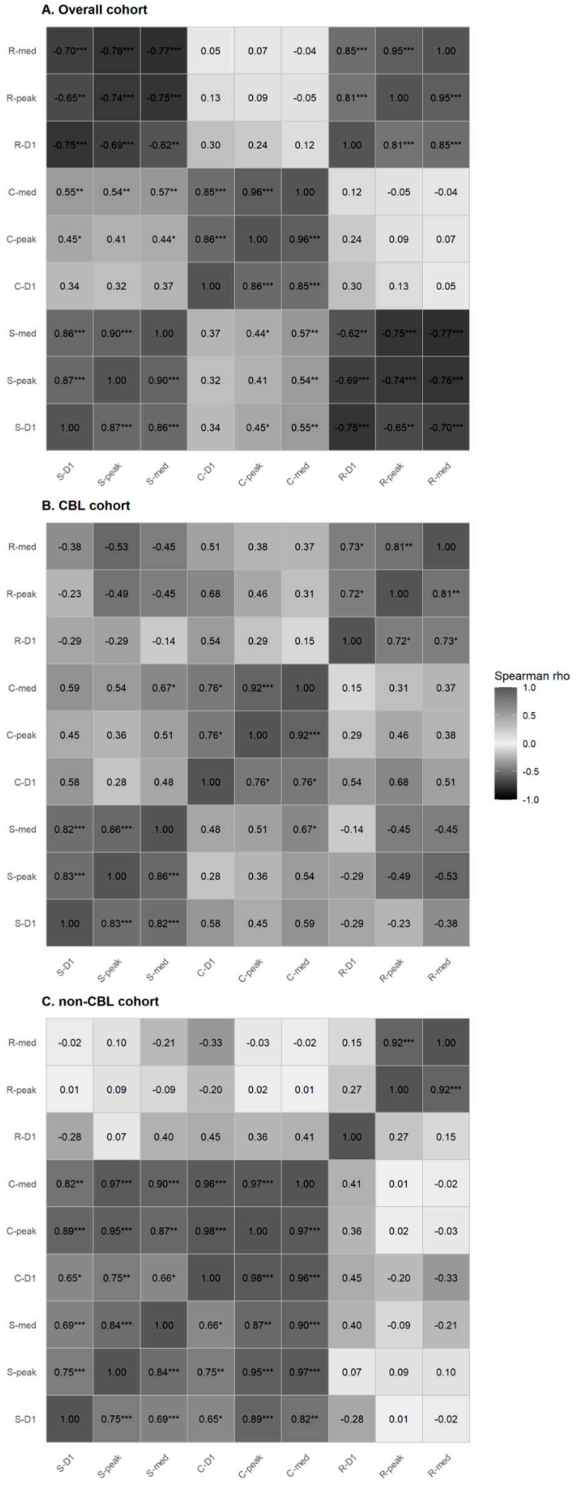

Panel A shows the overall cohort, Panel B the CBL cohort, and Panel C the non-CBL cohort. S-D1, serum S100B on day 1; S-peak, peak serum S100B; S-med, median serum S100B; C-D1, CSF S100B on day 1; C-peak, peak CSF S100B; C-med, median CSF S100B; R-D1, CSF-to-serum S100B ratio on day 1; R-peak, peak ratio; R-med, median ratio. Values are Spearman rho coefficients. Asterisks denote Benjamini–Hochberg-adjusted significance: *$p < 0.05$, **$p < 0.01$, ***$p < 0.001$. Peak and median indices required at least two serial measurements. Correlations involving ratio-derived indices should be interpreted cautiously because serum S100B is included in the denominator.
